# Editors’ Introduction: Understanding and Addressing Vaccine Hesitancy

**DOI:** 10.3390/vaccines13060613

**Published:** 2025-06-06

**Authors:** Gary L. Kreps, Xuewei Chen, Ming Li

**Affiliations:** 1 Center for Health & Risk Communication, George Mason University, Fairfax, VA 22030, USA; 2 School of Community Health Sciences, Counseling and Counseling Psychology, Oklahoma State University, Stillwater, OK 74078, USA; xuewei.chen@okstate.edu; 3 Department of Health Sciences, Towson University, Towson, MD 21252, USA; mli@towson.edu

## 1. Background

The goal of this Special Issue, “Understanding and Addressing Vaccine Hesitancy”, is to examine the current state of knowledge concerning the major causes and influences of vaccine hesitancy, as well as to suggest evidence-based solutions for the growing problem of vaccine hesitancy in modern society. The 11 scientific articles selected through rigorous peer review for publication in this Special Issue carefully examine, through the use of unique and robust research designs, the complex issues related to how vaccine hesitancy arises among a range of consumer populations across the globe to influence vaccination adherence for dangerous health problems such as COVID-19, childhood diseases, influenza, HPV, HIV/AIDS, and several other challenging health risks. The studies included in this Special Issue examine the key factors that lead to vaccine hesitancy and its influence on important health outcomes, and describe how their results suggest relevant strategies for minimizing vaccine hesitation. It is our sincere hope that these important research articles provide relevant data that help document how vaccine hesitancy can lead to unequal participation in vaccination programs by key (often vulnerable) populations and the inequitable distribution of vaccines within and across nations; chronicle how the negative influences of disinformation, misinformation, and conspiracy theories on health communication have encouraged vaccine hesitancy; and also highlight how effective vaccine promotion and intervention programs, policies, and communication strategies can be designed and implemented to counter vaccine information avoidance, health information overload, and distrust in science to enhance the delivery of important life-saving vaccinations to at-risk populations.

## 2. Collection of Special Issue Articles

For example, the first article in the Special Issue, by Zhang et al., “Combating the Co-Circulation of SARS-CoV-2 and Seasonal Influenza: Identifying Multi-Dimensional Factors Associated with the Uptake of Seasonal Influenza Vaccine among a Chinese National Sample”, utilizes a cross-sectional online survey conducted on a large representative national quota sample of 3161 Chinese adults from across different regions of China to examine how key multi-dimensional factors, such as knowledge of seasonal influenza, health perceptions, cues to action, patient–provider relationships, and COVID-19 pandemic-related issues influenced the uptake of seasonal influenza vaccines across China. The survey results provided clear recommendations for designing and implementing health promotion programs and campaigns that address these complex issues to reduce influenza vaccine hesitancy and increase much-needed vaccination in China.

The second article in the Special Issue, by Furlan et al., “The Impact of Age and Vaccine Conspiracy Beliefs on COVID-19 Vaccine Uptake among United States Adults”, uses survey data to provide important insights into the sociodemographic characteristics of adults in the US who are most likely to, firstly, believe negative COVID-19 vaccine conspiracy theories and, secondly, resist getting vaccinated. For example, the study found that political conservatism was a particularly strong predictor of both vaccine hesitancy outcome variables. However, they also learned that age, income, and education were additional important demographic factors involved in the belief of negative vaccine conspiracy theories. These finding suggest important strategies for designing strategic vaccination promotion education campaigns that effectively reach and influence major at-risk demographic segments of the population who are most likely to accept and be influenced by anti-vaccination conspiracy theories.

The third article in the Special Issue, by Domaradzki et al., “Investigating Beliefs in Anti-Vax Conspiracy Theories among Medical Students”, builds upon the second article’s examination of how anti-vaccine conspiracy theories influence vaccine hesitancy by exploring how these conspiracy theories influence medical students, a crucial emergent healthcare provider population for guiding public decisions related to vaccine hesitancy. This study collected survey data from 441 medical students to explore their attitudes toward six of the most prevalent anti-vaccine conspiracy theories. Interestingly, a surprising finding was that a relatively large proportion of this healthcare provider population held ambivalent attitudes toward anti-vaccine conspiracy theories, with 20% of the medical students expressing belief in or being unsure about at least one of these conspiracy theories. The study indicates that it is questionable how well these healthcare providers will be able to encourage vaccination among their patients with these kinds of ambivalent beliefs. A strong issue raised by this study is that medical students need to be provided with strong educational programs concerning the importance of vaccination to help their current and future patients combat vaccine hesitancy based on anti-vaccine conspiracy theories.

The fourth article in the Special Issue, by Degarege et al., “Evaluation of Theoretical Frameworks to Detect Correlates of HPV Vaccination in the Midwest, US, Using Structural Equation Modeling”, conducted a cross-sectional survey of 1306 teenagers and young adults in the Midwest, US, to test how well the Integrated Health Theory (IHT), which combines important elements of the Health Belief Model, Theory of Planned Behavior, Theory of Reasoned Action, Social Cognitive Theory, and Socioecological Theory, predicted the reasons for HPV vaccine hesitancy that were expressed by members of this population. The study found a good conceptual fit between the key factors in the IHT model and the survey responses concerning the specific beliefs, attitudes, and norms that influenced the willingness to receive the HPV vaccine by these teenagers and young adults. This study’s findings suggest utility in using IHT to guide HPV vaccine promotion interventions with members of this population.

The fifth article in the Special Issue by Chen et al., “Identifying Mental Health Literacy as a Key Predictor of COVID-19 Vaccination Acceptance among American Indian/Alaska Native/Native American People”, explores how two critically important communication factors, health literacy and mental health literacy, influence COVID-19 vaccine acceptance among members of a particularly vulnerable minority population in the US: American Indians/Alaska Natives/Native Americans. While health literacy refers to the ability to find, understand, and use relevant health information, mental health literacy refers to the knowledge, attitudes, and skills needed to promote and manage mental health wellbeing. Past research has suggested that both health literacy and mental health literacy are related to COVID-19 vaccine uptake, but this has not been studied particularly well in the past in this minority population. The study found that both the health literacy and mental health literacy were strong predictors of the willingness to receive the COVID-19 vaccine, suggesting the need to promote both health literacy and mental health literacy to help overcome problems with vaccine hesitancy among this vulnerable population.

The sixth article in the Special Issue, by Del Ducca et al., “Risk Awareness as a Key Determinant of Early Vaccine Uptake in the Mpox Vaccination Campaign in an Italian Region: A Cross-Sectional Analysis”, reports the results of an anonymous online survey responded to by 1717 subjects who received MPOX vaccinations to examine the vaccine hesitancy factors that led to the early acceptance of the vaccine in response to an enrollment campaign conducted in the Lazio region of Italy (less than 30 days from the vaccine campaign start date) and late vaccination acceptance (more than 30 days after the start of the vaccine campaign). They found that most of the respondents were early vaccine acceptors (92.5%), compared to only 7% who were late vaccine adopters. However, there were some interesting differences between these two groups. For example, the early vaccine adopters were likely to possess a high degree of risk awareness concerning MPOX infection, while the late adopters reported lower levels of education and lower perceptions about their physical and mental health status. These findings suggest the need for targeted health education campaigns to reduce MPOX vaccine hesitancy for members of the delayed vaccine acceptance group to promote awareness about their risks of MPOX infection and to help them understand how the vaccine could improve their health outcomes.

The seventh article in the Special Issue, by Iova et al., “Knowledge, Attitudes, Intentions and Vaccine Hesitancy among Postpartum Mothers in a Region from the Northwest of Romania”, examined the factors that influenced Romanian mothers during their postpartum period to be hesitant about accepting childhood vaccinations for their newborn children. Based upon 404 mothers’ responses to the Vaccine Hesitancy Identification Survey, the researchers identified and compared two groups of mothers: the pro-vaccine group, who were not hesitant, and the non-vaccine group, who were hesitant to have their children vaccinated. They found that the non-vaccine group had low levels of knowledge and information regarding childhood vaccination, had high concerns about adverse reactions to the vaccine, and were more focused on the risks related to the vaccine than on the benefits from vaccinating their children. These findings suggest the strong need for implementing information campaigns to help increase vaccine-hesitant mothers’ knowledge about the benefits and safety of childhood vaccination in Romania.

In the eighth article in the Special Issue, “It Doesn’t Cure, but It Protects”: COVID-19 Vaccines through the Eyes of Children and Their Parents”, Groenewald et al. conducted a qualitative interview study exploring the experiences of a paired sample of South African children (under the age of 18) and their parents concerning the COVID-19 vaccine between 2020 and 2021 to examine the intergenerational influences within families on vaccine acceptance and hesitancy. Their findings showed a moderately high level of vaccine acceptance among most of the children and their parents, illustrating optimism that the vaccine would help them withstand the COVID-19 virus. Importantly, they found that those parents who expressed acceptance of the vaccine strongly influenced their children’s confidence in the vaccine, while those parents who were unsure about the vaccines encouraged vaccination hesitancy reactions from their children. They also found that vaccine hesitancy among both parents and children was related to their limited vaccine literacy. These findings strongly illustrate the importance of vaccine education efforts to increase vaccine literacy among both parents and their children.

The ninth article in the Special Issue by Tolley et al., “The Moderating Effect of Vaccine Hesitancy on the Relationship between the COVID-19 Vaccine Coverage Index and Vaccine Coverage”, reported a secondary analysis of publicly available county-level data collected in the US that was administered by the US Department of Health and Human Services and the US Centers for Disease Control and Prevention in their Household Pulse Survey (HPS) to examine the levels of county-level vaccination and vaccine hesitancy, in order to establish a COVID-19 Vaccine Coverage Index (CVAC), which identifies social and structural barriers to vaccine coverage across five categories: sociodemographics, resource-constrained health systems, healthcare accessibility, irregular care-seeking behavior, and historic under-vaccination. They then compared racial and ethnic segments of the population to evaluate how vaccine hesitancy moderated vaccine acceptance, finding that the vaccine coverage was stronger in low-hesitancy counties compared to that in the high-hesitancy counties. They also found that counties with low levels of social/structural barriers (CVAC scores) but high levels of vaccine hesitancy had lower levels of vaccine coverage. Interestingly, when they introduced into their analysis county-level ethno-racial composition rates, they found that counties with higher proportions of white residents significantly predicted decreased vaccination rates. These findings indicate that CVAC scores can be effectively combined with an analysis of vaccine hesitancy measures in predicting vaccine uptake and to identify the most relevant segments of the population to target for vaccine promotion interventions.

The tenth article in the Special Issue, by Desens et al., “A Comparative Case Study Analysis: Applying the HIPE Framework to Combat Harmful Health Information and Drive COVID-19 Vaccine Adoption in Underserved Communities”, examined how an innovative new model, the HIPE (Health Information Persuasion Exploration) framework, could be used to evaluate two health campaign case studies. The campaigns examined were designed to address COVID-19 vaccine hesitancy in two unique at-risk communities (a Black/Haitian community in Miami, Florida, and a migrant agricultural worker community in Central Valley, California), in which both campaigns achieved their vaccination promotion goals. The HIPE framework examines how persuasive communication strategies are used in four key vaccine promotion campaign areas (detect, analyze, design, and evaluate) to influence vaccination acceptance and self-efficacy as a holistic, data-driven approach to evaluate how campaigns examine and respond to the ways that harmful information exposure influences vaccination acceptance. Their comparative case study analysis indicated that the HIPE model provided robust insights into how the detection of and response to inaccurate/misleading health information informed unique campaign messaging to promote vaccination in both cases examined, suggesting the strong need to develop effective community-specific precise intervention communication strategies to combat vaccine hesitancy.

The eleventh and final research article in the Special Issue, by Nwachukwu et al., “Understanding COVID-19 Vaccine Hesitancy in the United States: A Systematic Review”, reported an in-depth systematic review of 544 research studies that examined the factors contributing to COVID-19 vaccine hesitancy, including an analysis of the perceptions of vaccine confidence, disease risk complacency, individual benefit calculations, the convenience of the vaccine, and evaluations of the responsibility to protect others with vaccination. This insightful review identified major issues that influence vaccine hesitancy, including the strong influences of concerns over vaccine safety, and suggests the efficacy of using transparent and effective communication, such as the use of tailored messaging strategies that are responsive to unique community socioeconomic and cultural audience issues, along with the implementation of active efforts to promote community engagement to overcome hesitancy and promote vaccine uptake.

## 3. Discussion

Combined, the eleven research studies published in this Special Issue collectively provide important insights into the nature of vaccine hesitancy across different, geographically dispersed, culturally unique, and socioeconomically diverse populations. The studies introduce important new methods for examining vaccine hesitancy, evaluating the unique factors leading to vaccine hesitancy in different communities, and suggest important new intervention evaluation and planning strategies for overcoming vaccine hesitancy and promoting vaccine acceptance and uptake. While there is clearly still much to learn about the nature of vaccine hesitancy and there will be many new challenges to vaccine acceptance that will inevitably arise as we confront new societal health risks, this Special Issue provides fruitful directions for guiding future vaccine promotion research and intervention.

